# Uncovering the Internal Structure of the German Version of the CORE‐OM: A Network Analysis

**DOI:** 10.1002/cpp.70063

**Published:** 2025-03-19

**Authors:** Jürgen Fuchshuber, Marina Zeldovich, Gabor Aranyi, Lisa Winter, Martin Kuska, Dominique Dumont, Elke Humer, Human‐Friedrich Unterrainer

**Affiliations:** ^1^ Department of Psychoanalysis and Psychotherapy Medical University Vienna Vienna Austria; ^2^ Comprehensive Center for Clinical Neurosciences and Mental Health Medical University Vienna Vienna Austria; ^3^ Center for Integrative Addiction Research (CIAR) Grüner Kreis Society Vienna Austria; ^4^ Faculty of Psychotherapy Science Sigmund Freud University Vienna Austria; ^5^ Institute of Psychology University Innsbruck Innsbruck Austria; ^6^ Institute of Education and Psychology at Szombathely ELTE Eötvös Loránd University Budapest Hungary; ^7^ Department for Psychosomatic Medicine and Psychotherapy University for Continuing Education Krems Krems an der Donau Austria; ^8^ Institute of Psychology University of Graz Graz Austria; ^9^ University Clinic for Psychiatry and Psychotherapeutic Medicine Medical University Graz Graz Austria; ^10^ Department of Religious Studies University of Vienna Vienna Austria

**Keywords:** confirmatory factor analysis, CORE‐OM, exploratory graph analysis, factorial validity, network analysis, patient‐reported outcomes, psychometrics

## Abstract

**Background:**

The Clinical Outcomes in Routine Evaluation – Outcome Measures (CORE‐OM) is a pantheoretical diagnostic instrument that has been widely used in mental health research. Nevertheless, the exploration of the factor structure of the CORE‐OM yields diverse results.

**Aims:**

This study aimed to explore the internal structure of the German CORE‐OM using network analysis and compare several competing factorial structures of the CORE‐OM with traditional confirmatory factor analysis (CFA) to gain a more comprehensive understanding of its structural validity.

**Method:**

A total sample comprised 4496 (63% female) participants from an outpatient population. In a first step, we used network analysis (*n*_1_ = 2248) to assess relationships between the items, followed by explorative graph analysis (EGA) to analyse community structure. Finally, we specified five competing models, including the one derived from the EGA, and used CFA in a second sample (*n*_2_ = 2248) to identify the best‐fitting structure of the instrument.

**Results:**

The estimated cross‐sectional network demonstrated high correlation stability. The average item predictability was *R* ^2^ = 0.42. The EGA identified four distinct communities in the German CORE‐OM (General Problems; Interpersonal Problems; Positive Resources; Self Harm Risk). Confirmatory factor analysis showed that the EGA‐derived models had the most parsimonious fit.

**Conclusions:**

These findings suggest a refined structure for the CORE‐OM, highlighting key item relationships and offering potential improvements for scoring and clinical use.

**Summary**

- The study highlights the utility of network analysis and CFA in uncovering complex relationships between items, suggesting these methods as valuable tools for improving psychometric instruments.
- The refined scoring model offers potential improvements for clinical practice, allowing for more accurate identification of patient needs and tailored interventions.
- Inter‐item relationships suggest that a revised version of the CORE‐OM could better capture the nuances of mental health problems, thereby enhancing its diagnostic and therapeutic utility.

## 1 Introduction

### 1.1 The CORE‐OM

The Clinical Outcomes in the Routine Evaluation – Outcome Measure (CORE‐OM; (Evans et al. 2002)) is a 34‐item self‐report questionnaire designed to measure psychological distress and risk behaviours in counselling and therapy settings. The CORE‐OM was developed in the early 2000s to provide a standardised outcome system for practice‐based evidence in psychological therapies (Evans et al. 2002) and is widely used due to its ease of administration, free availability, and multilingualism. There are currently up to 50 translations into different languages and dialects (Zeldovich and Alexandrowicz 2019), enabling not only data collection and screening of patients but also comparisons between countries and cultures.

However, CORE‐OM does have ambiguities regarding factorial validity, which arose in the original English version and have subsequently been reproduced in its translations. In particular, the original theoretical four‐factor structure with four domains (functioning: 12 items, problems/symptoms: 12 items, well‐being: four items, and risk: six items) did not provide desirable fit indices in confirmatory factor analyses. Instead, an exploratively derived three‐component solution based on item valence (positively and negatively worded items, and risk) has repeatedly been found to best describe the data in both studies of the original English CORE‐OM (e.g. Evans et al. 2002; Handscomb et al. 2016) and its translations (Zeldovich and Alexandrowicz 2019). In addition, some studies suggested alternative, more complex (bi‐) factorial structures with more limited applicability to clinical practice (Deng et al. 2024; Falkenström et al. 2018; Lyne et al. 2006). A recent investigation of the factorial validity of the CORE‐OM in a large outpatient sample (Aranyi et al. Under review) found a three‐component solution to fit the data best, forming scales assessing resources (positively worded items), stress burden (negatively worded items) and risk (risk items plus the item 26 ‘I thought I have no friends’), consistent with the model derived from the nonclinical sample in the original English study (Evans et al. 2002). With more than two decades of attempts to find a suitable factorial structure for the CORE‐OM, and no alternatives yet proposed for clinical implementation, there is still a need for further investigation into this topic. A recent study of the factorial structure of the Chinese CORE‐OM (Deng et al. 2024) has already introduced initial findings from the network approach, which provides a promising tool for investigating other CORE‐OM translations using the same analytical framework. In the following, we briefly outline the advantages of this approach as a contribution to a better understanding of factorial validity of the German version of the instrument.

Network analysis offers several advantages over traditional validation methods in understanding complex psychological constructs, particularly in investigating the interactions between symptoms (Borsboom and Cramer 2013). Unlike traditional methods, like principal component analysis (PCA), which aims to identify underlying factors by extracting components that explain the maximum variance in the data (Jolliffe and Cadima 2016), network analysis provides a more dynamic perspective. PCA transforms data into a set of linearly uncorrelated variables called principal components, which can highlight clusters or scales by analysing how items load onto these components (Jolliffe and Cadima 2016). This method is effective in summarising data and uncovering latent structures, but it assumes that the variance among items can be explained by a smaller number of underlying factors (Wang 2009).

In contrast, network analysis conceptualises psychological constructs as systems of interacting components. In this framework, individual symptoms or items are viewed as ‘nodes’ within a network, and the relationship between them, or ‘edges’, represent direct (or ‘conditioned’) interactions. Based upon these interrelationships, community detection methods are able to identify clusters of similar nodes. These groups of interconnected nodes, referred to as ‘communities’, are mathematically similar to factors identified in traditional models like PCA (Golino and Demetriou 2017). This approach offers several advantages. First, network analysis can identify key nodes that are central to the network, determine the strength of connections between items and detect communities or clusters. Moreover, a recent development in this field, exploratory graph analysis (EGA), allows for the exploration and identification of these communities, further enhancing the understanding of the structure and dynamics of psychological constructs (Borsboom and Cramer 2013; Epskamp, Borsboom, and Fried 2018).

EGA can help to capture the complexity of item relationships more so than traditional explorative methods like PCA. This superiority stems from the difference in approach: PCA isolates the factor that explains the most variance and removes its effect in a linear sequence, while EGA builds item patterns from the ground up, using the entire dataset via regularised regression (Golino and Epskamp 2017). Moreover, EGA outperforms conventional dimension‐reduction techniques by providing more reliable parameter estimates, as shown in simulation studies and real world psychometric data (Forkmann et al. 2018; Golino and Demetriou 2017; Golino and Epskamp 2017). Finally, it usually offers a clearer, more interpretable structure of symptom dimensions, thanks to its graphical representation, which highlights the connections between nodes both within and across dimensions.

In summary, while traditional methods such as PCA are useful for summarising data and identifying latent factors, a more flexible, simplified, and data‐driven approach is useful to uncover the often complicated relationships and structures within psychological constructs.

#### 1.1.1 Study Aims

The study contributes to the ongoing discussion on the factorial validity of the CORE‐OM, particularly its German version, by leveraging the advantages of network analysis. We aim to provide a more refined understanding of the instrument's factor structure that could inform its clinical application and further development.

Specifically, our objectives are:

- To explore the underlying structure of the German CORE‐OM by identifying the most central items and examining the direct relationships between them within a network framework.
- To assess the predictability of individual items within the network, offering a better understanding of how well specific symptoms or behaviours can be anticipated based on their connections within the overall structure.
- To identify and analyse clusters of items within the CORE‐OM network using community detection methods. By doing so, we aim to uncover potential areas where different psychological constructs or domains group together, providing insights that could enhance the instrument's clinical utility.
- To compare the findings from network analysis with competing factorial structures of the CORE‐OM using traditional confirmatory factor analysis (CFA), revealing the most fitting factor structure of the instrument.

## 2 Material and Methods

### 2.1 Sample and Procedure

Our study included a total of 4496 participants (63% female). Data was collected from May 2014 to March 2024. Participants were outpatients of the psychotherapeutic clinic of Sigmund Freud University Vienna (SFU; Salztorgasse, Vienna, Austria), who had completed the CORE‐OM during their intake process. The outpatient clinic of SFU provides a wide range of psychotherapeutic services within a dynamic academic environment, catering to a diverse clientele, coming from a variety of cultural backgrounds. As part of its commitment to integrating research and practice, the clinic has established a standardised intake process that includes the administration of the CORE‐OM to all incoming clients. This approach ensures comprehensive data collection that supports both clinical assessment and ongoing research. To obtain a dataset suitable for network analysis and confirmatory factor analysis, only patients who had completed the CORE‐OM with no missing responses were included. Informed consent was obtained from all patients before they began the questionnaire. The research adhered to the Declaration of Helsinki and the protocol was approved by the Ethics Commission of the Sigmund Freud University, Vienna (Protocol Code: HCEXEKJGBDWDJI89466).

### 2.2 Psychometric Assessment

#### 2.2.1 Sociodemographic Information

After giving informed consent, participants completed a sociodemographic questionnaire. This included questions about age, gender, marital status, education level and area of study, current occupation or training, psychiatric disorders, medication use and country of origin and language proficiency.

#### 2.2.2 The CORE‐OM

The CORE‐OM consists of 34 items using a five‐point Likert‐type response scale (0 = ‘not at all’ to 4 = ‘most or all of the time’). The items are designed to assess distress, with higher scores indicating greater impairment. Eight positively worded items have to be reversed before calculating the domain and total scores. The German version of the CORE‐OM provides adequate reliability (Cronbach's *α* of 0.74 and McDonald's *ω* of 0.79 for the risk domain to *α* and *ω* of 0.92 for the total score) for all domains except the well‐being domain (*α* and *ω* of 0.68; Aranyi et al. Under review), which corresponds to the results of the original version (Evans et al. 2002).

### 2.3 Statistical Analyses

We used the SPSS 29.0 for data management and descriptive statistics. Network analysis and confirmatory factor analysis (CFA) was conducted in R version 4.4.1. Before the analysis, the total sample was split into two equally large groups via the randomised allocation function in SPSS. The network analysis applied to sample 1 utilised the extended Bayesian information criterion (EBIC) for graphical Lasso methodology (Chen and Chen 2008; Tibshirani 1996) with the corMethod = ‘cor_auto’ (Epskamp, Borsboom, and Fried 2018) and a hyperparameter *γ* = 0.5. The network estimation was computed using the qgraph package (Epskamp et al. 2012). Our network comprised 34 nodes. We utilised an adapted Fruchterman–Reingold algorithm for node placement (Fruchterman and Reingold 1991; Jones, Mair, and McNally 2018). To determine the key nodes within the network, we employed centrality indices calculated through the centrality function of the qgraph package. Besides the edge weights, we investigated the following measures: (1) expected influence (EI), which is the aggregate weight of edges a node has with every other node in the network and also considers negative links of variables (Robinaugh, Millner, and McNally 2016); (2) centrality strength (CS) corresponding to the absolute weights of all edges directly connected to the respective node (Bringmann et al. 2019); (3) closeness, which sums the average distances from a node to all other nodes in a network (Borgatti 2005). Furthermore, we assessed the predictability, which assesses the proportion of variance of a node explained by its adjacent nodes (Haslbeck and Waldorp 2018).

To assess stability of the network and the precision of our estimated parameters, we employed bootstrapping techniques (with 2000 bootstrap samples via the bootnet package (Epskamp and Fried 2018); version 1.4.3). To investigate the accuracy and robustness of the edge weights, we derived 95% confidence intervals (CIs) for each edge's estimated value. For the analysis of the stability of investigated network measures (EI, CS and bridge centrality), we utilised the correlation‐stability coefficient (CS coefficient), which measures how much of the dataset can be omitted while still ensuring a correlation of at least 0.7 with the original data, with 95% confidence. A CS coefficient above 0.50 is desirable for interpreting a network as stable, whereas a value below 0.25 suggests that the network's centrality order is unstable (Epskamp, Borsboom, and Fried 2018).

To investigate the underlying structure of the CORE‐OM, we employed exploratory graph analysis (EGA) using the EGAnet package (version 1.2.3) (Golino and Epskamp 2017). This method, grounded in network psychometrics, examines the direct relationships among the observed variables, or items on the CORE‐OM, by modelling their interactions within a network framework. Stability of the identified communities was assessed by bootstrap exploratory graph analysis (bootEGA, 1000 iterations). EGA employs the walktrap community detection algorithm to identify the underlying dimensions in network models. This algorithm estimates communities through the use of ‘random walks’ across the network (Pons and Latapy 2006). These random walks repeatedly cross neighbouring edges, with higher edge weights indicating more likely paths of travel. Starting from each node, the algorithm repeatedly takes steps—moving from one node to another across edges—until it forms a community boundary. A node's community is determined by its connection to many densely connected edges and fewer sparsely connected ones. The algorithm operates deterministically, meaning that it identifies the number and structure of the communities independently, without requiring input or guidance from the researcher.

Seven competing CORE‐OM models (original four‐domain structure, single higher order factor structure, three‐factor structure, two EGA‐derived four‐factor structure models by Deng et al. [2024]) and the present study, as well as two additional bifactor versions of the EGA models, were assessed with the robust weighted least squares (WLSMV) estimator for ordinal data as well as the robust maximum likelihood (MLM) estimator for continuous data in order to assure comparability to previous studies using lavaan package (Rosseel 2012). Following Kline (2015), the following cutoff values for acceptable global fit indices for the WLMSV‐estimator analysis were applied: (1) Tucker–Lewis index relative fit index (TLI); normed fit index (NFI), comparative fit index (CFI) > 0.90; (2) the square root error of approximation (RMSEA) < 0.08 and the upper bound of its 90% confidence interval < 0.10. Finally, in order to compare the models regarding their parsimoniousness, we further assessed the Akaike information criterion (AIC) with the MLM estimator. Generally, the model with a lower value indicates a more parsimonious model (Vrieze 2012).

The code used in our analysis is available in the Supplements.

## 3 Results

### 3.1 Sample Characteristics and Descriptives

The detailed descriptive characteristics of the sample are presented in Table S1. The data analysis included a total of 4496 individuals who completed the CORE‐OM. Both samples were predominantly composed of German‐speaking individuals from Germany, Austria and Switzerland, accounting for 64.1% of participants (German as first language). 35.9% stated that they had sufficient knowledge of German to complete the questionnaire. Age ranged from 18 to 81 years in the EGA sample (*M* = 30.41; SD = 11.03) and between 18 and 80 in the CFA sample (*M* = 31.01; SD = 10.48). A majority of participants (62.1% and 63%) identified as female. The majority had at least a high school education. The most common primary diagnosis in the both samples was neurotic stress–related and somatoform disorders (F40–F48; 37.2% and 38.5%, respectively). Most participants of both groups were diagnosed with at least one psychiatric disorder (66.7% and 65.6%). Finally, 26.1% in the EGA sample and 24.8% in the CFA sample were diagnosed with at least one comorbid disorder. Detailed items are displayed in Table S2.

### 3.2 Network Analysis

#### 3.2.1 Network Stability

As highlighted in Figure S1, the application of a case‐dropping bootstrap technique to evaluate centrality stability yielded a CS coefficient of 0.75 for the EI centrality and strength centrality. Hence, both centrality parameters showed an acceptable degree of stability. Of note, the bootstrap analysis for edge weights demonstrated that the edges were consistently stable, evidenced by the relatively narrow confidence intervals (see Figure S2). In sum, the network exhibited high levels of accuracy and stability, making it suitable for analysis.

#### 3.2.2 Network Estimation

Figure S3 visualises the regularised EBICglasso network plot. As shown, the CORE‐OM items exhibited an overall dense network with generally positively correlated relationships between them. This is with the exception of the relationship between Items 5 and 6, as well as 4 and 22, which exhibited negative edges.

#### 3.2.3 Network Inference

As highlighted in Figure S4, Item 23 (felt despairing or hopeless) and 17 (overwhelmed by problems) were found to exhibit the highest EI within the CORE‐OM (see Figure S5). The least influential indicators were item 20 (problems impossible to put to one side), eight (troubled by aches/pains), 19 (felt warmth/affection) and 30 (to blame for problems). Furthermore, while Item 6 showed a rather low EI, the simultaneous high strength underscores its partially inhibiting effect in relation to the total network. Generally, a similar pattern was observed for centrality strength; however, bootstrap difference tests revealed a less clear pattern regarding significant differences (see Figure S6 for the results of the bootstrap difference tests).

The average node predictability by all other nodes of the network was *R* ^2^ = 0.40. The node with the highest predictability was Item 23 (*R* ^2^ = 0.66). Item 8 had the lowest predictability (*R* ^2^ = 0.16). The network plot highlights the predictability scores as pie charts around each node (see S4).

#### 3.2.4 Exploratory Graph Analysis

**FIGURE 1:**
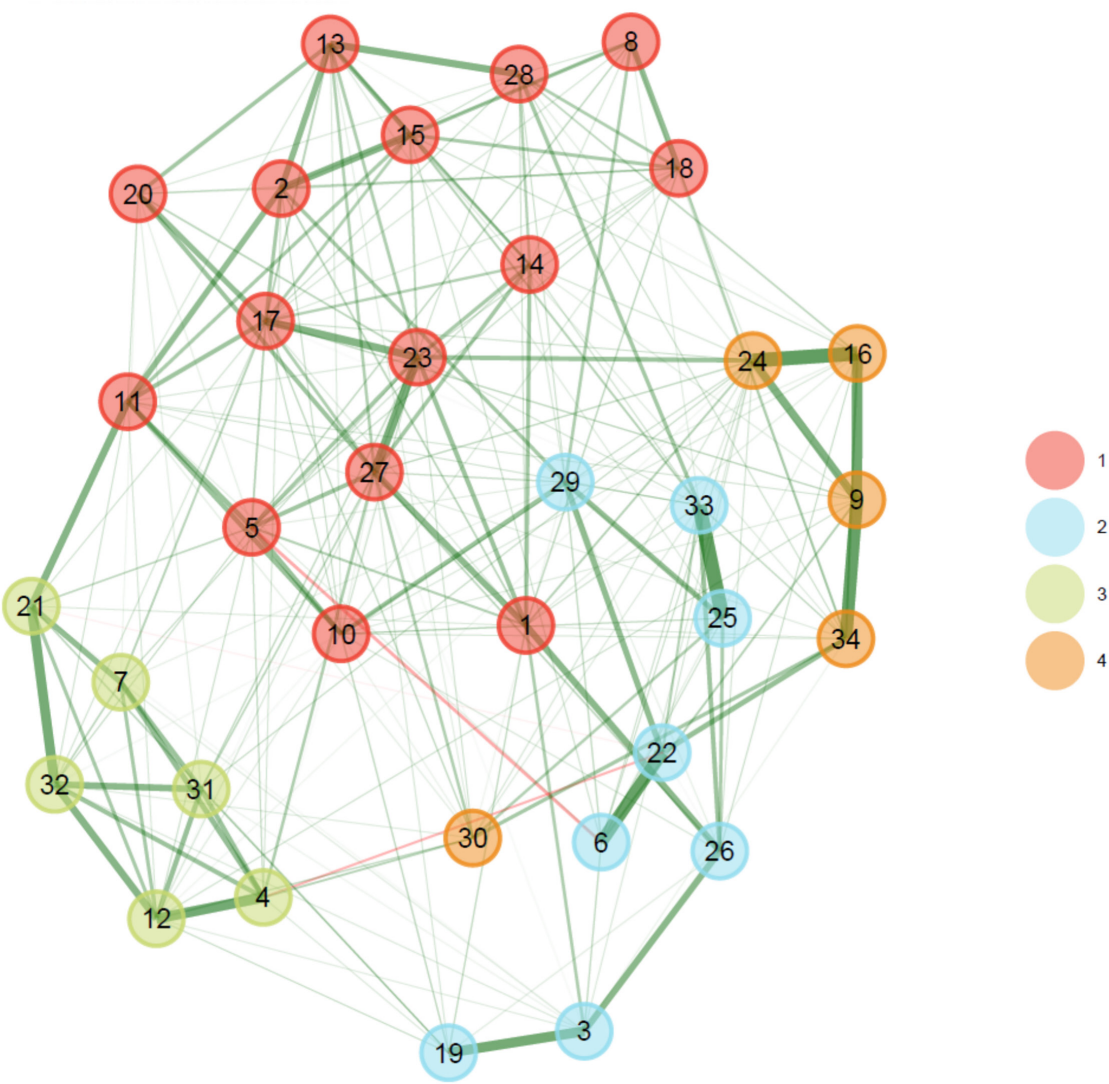
Explorative graph analysis of the CORE‐OM. *Note*: *N* = 2248; numbers denote empirical communities. 1 = general problems; 2 = interpersonal problems; 3 = positive resources; 4 = self‐harm risk.

Figure 1 depicts the results of the explorative graph analysis. The results suggest the following four communities (for details on item labels, see Table 1, column EGA‐derived four‐factor [present study]):

**TABLE 1:** Comparative list of item‐scale allocation.

No.	Item (shortened version)	Original four domains (Evans et al. 2002)	Three factors (Zeldovich and Alexandrowicz 2019)	EGA‐derived four‐factor (Deng et al. 2024)	EGA‐derived four‐factor (present study)
1	Alone and isolated	Functioning	Negative	General problems	General problems
3	Someone to turn to for support (+)	Functioning	Positive	Positive resources	Interpersonal problems
7	Able to cope when things go wrong (+)	Functioning	Positive	Positive resources	Positive resources
10	Talking too much for me	Functioning	Negative	Problems with others	General problems
12	Happy with things done (+)	Functioning	Positive	Positive resources	Positive resources
19	Felt warmth/affection (+)	Functioning	Positive	Positive resources	Interpersonal problems
21	Done most things needed to (+)	Functioning	Positive	Positive resources	Positive resources
25	Felt criticised by others	Functioning	Negative	Problems with others	Interpersonal problems
26	Thought I have no friends	Functioning	Risk	Problems with others	Interpersonal problems
29	Irritable with other people	Functioning	Negative	Problems with others	Interpersonal problems
32	Achieved things wanted to (+)	Functioning	Positive	Positive resources	Positive resources
33	Felt humiliated or shamed	Functioning	Negative	Problems with others	Interpersonal problems
2	Tense, anxious, nervous	Problems	Negative	General problems	General problems
5	Lacking in energy/enthusiasm	Problems	Negative	General problems	General problems
8	Troubled by aches/pains	Problems	Negative	General problems	General problems
11	Tension/anxiety prevented	Problems	Negative	General problems	General problems
13	Disturbed, unwanted thoughts	Problems	Negative	General problems	General problems
15	Felt panic or terror	Problems	Negative	General problems	General problems
18	Difficulty sleeping	Problems	Negative	General problems	General problems
20	Problems impossible to put to one side	Problems	Negative	General problems	General problems
23	Felt despairing or hopeless	Problems	Negative	General problems	General problems
27	Felt unhappy	Problems	Negative	General problems	General problems
28	Images/memories disturbing	Problems	Negative	General problems	General problems
30	To blame for problems	Problems	Negative	General problems	Self‐harm risk
6	Physically violent to others	Risk	Risk	Risk	Interpersonal problems
9	Thought of hurting myself	Risk	Risk	Risk	Self‐harm risk
16	Made plans to end my life	Risk	Risk	Risk	Self‐harm risk
22	Threatened/intimidated by someone	Risk	Risk	Risk	Interpersonal problems
24	Better if dead	Risk	Risk	Risk	Self‐harm risk
34	Hurt self physically	Risk	Risk	Risk	Self‐harm risk
4	OK about myself (+)	Well‐Being	Positive	Positive resources	Positive resources
14	Felt like crying	Well‐Being	Negative	General problems	General problems
17	Overwhelmed by problems	Well‐Being	Negative	General problems	General problems
31	Optimistic about future (+)	Well‐Being	Positive	Positive resources	Positive resources

*Note:* (+): positively worded items, No: order of appearance of items in the questionnaire. Same colours highlight similar constructs assessed by factorial structures.

Community 1 (general problems): Items 1; 2; 5; 8; 10; 11; 13; 14; 15; 17; 18; 20; 23; 27; 28. Community 2 (interpersonal problems): Items 3; 6; 19; 22; 25; 26; 29; 33. Community 3 (positive resources): Items 4; 7; 12; 21; 31; 32. Community 4 (self‐harm risk): Items 9; 16; 24; 30; 34.

Figure S7 visualises the replication indices regarding the community detection of the individual items. In summary, item stability was generally high, with mean scores ranging from 1 to 0.81. (Community 1: 0.93–0.60; Community 2: 0.88–0.72; Community 3:1; Community 4: 1–0.42). The least reliably replication was observed for Items 30 (Community 4: 0.42) and 10 (Community 1: 0.60).

Table 1 provides a comparative listing of the different CORE‐OM models including the one derived by our EGA results.

#### 3.2.5 Structural Equation Modelling

**FIGURE 2:**
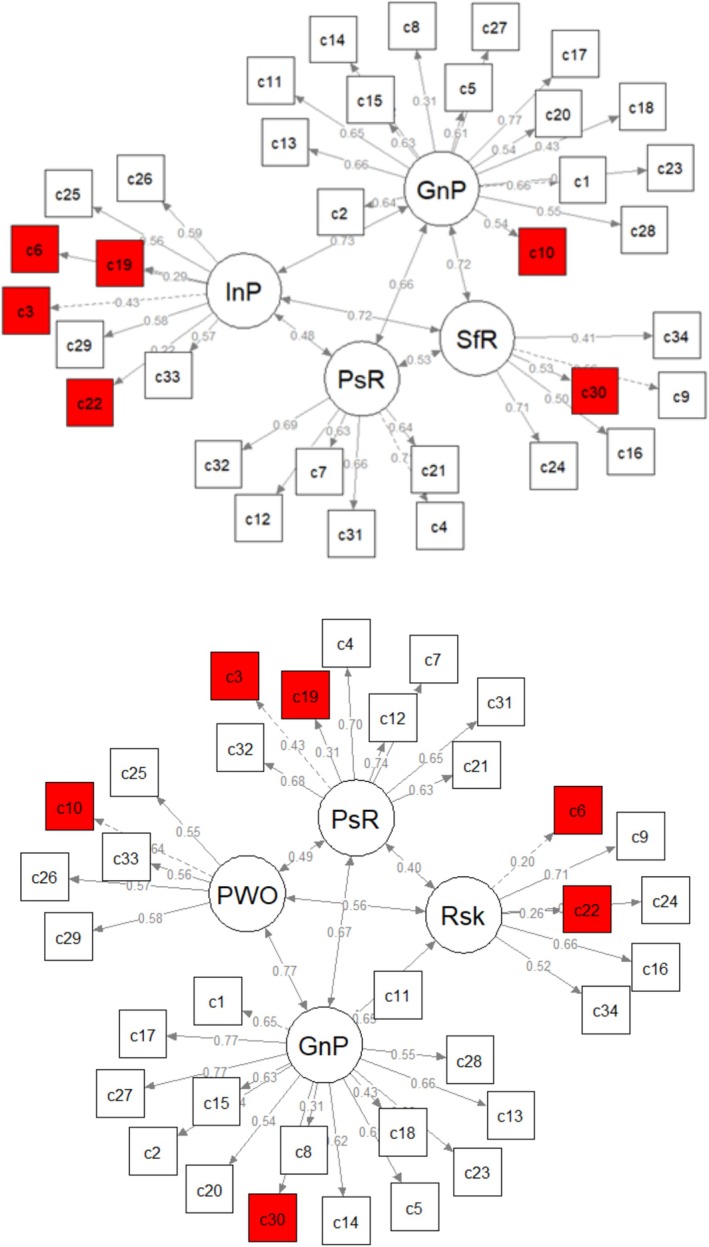
Comparison of structural equation models derived by EGA by the current study (above) and by Deng et al. (2024 visualised via the Fruchterman–Reingold algorithm. *Note*: *N* = 2248; GnP, general problems; InP, interpersonal problems; PsR, positive resources; PWO, problems with others; Rsk, risk; SfR, self‐harm risk. Differences in item allocation are highlighted in red.

In the next step, we estimated seven competing factorial structures of the CORE‐OM via CFA. The models were derived from previous research as well as the results of the EGA. Global fit indices are detailed in Table 2. None of the tested bifactor models converged using the weighted least squares (WLSMV) estimator, and thus, there excluded from further analysis. All other investigated models showed excellent fit indices under this condition. Based on the MLM‐derived AIC difference scores, both EGA‐derived four‐factor models showed a more parsimonious structure than the other models, with the model suggested by Deng et al. (2024) exhibiting the most parsimonious structure. Figure 2 visualises both EGA models with the Fruchterman–Reingold algorithm. As shown in Table S3, reliabilities for the factor structure ranged between acceptable (interpersonal problems: *α* = 0.65) to excellent (general problems: *α* = 0.90) for the EGA‐derived model of the present study. In comparison, the model derived by Deng and colleagues showed a similar pattern with Cronbach's *α* ranging from *α* = 0.71 (problems with others) to *α* = 0.89 (general problems).

**TABLE 2:** Confirmatory factor analysis fit statistics for the CORE‐OM.

Model	*χ* ^2^ (df)	*χ* ^2^ (df)	RMSEA (90% CI)	CFI	NFI	TLI	CFI (MLM)	AIC (MLM)
Original four domains (Evans et al. 2002)	4077.13 (521)	7.83	0.055 (0.053–0.057)	. 96	0.95	0.96	0.80	211479.98
Single higher order factor	4115.25 (523)	7.87	0.055(0.053–0.057)	0.96	0.95	0.96	0.79	211510.75
Three‐factor (Zeldovich and Alexandrowicz 2019)	2695.36 (524)	5.14	0.043 (0.041–0.045)	0.98	0.97	0.97	0.86	209744.48
EGA‐derived four‐factor (Deng et al. 2024)	2215.68 (489)	4.53	0.040 (0.038–0.041)	0.98	0.97	0.98	0.87	203941.38
EGA‐derived four‐factor (present study)	2763.93 (521)	5.31	0.044 (0.042–0.046)	. 97	0.97	0.97	0.86	209573.19

*Note:* *N* = 2248.

## 4 Discussion

Our study investigated a cross‐sectional network of the German version of the CORE‐OM, comparing it with traditional and previously suggested factorial structures using CFA. We aimed to contribute to the ongoing debate regarding the optimal structure of the instrument and to explore whether network analysis offers insights that can enhance the psychometric precision of the instrument and enable a more differentiated analysis of its various subdimensions.

The EGA revealed four distinct communities within the CORE‐OM items, each representing different aspects of the instrument's underlying structure. Community 1 encompasses items related to general stress and burden, with high replication indices (0.60 to 0.93) demonstrating a stable and reliable clustering of these items. Community 2 (RI = 0.72–0.88) seems to be especially connected to interpersonal problems. Community 3 groups positively worded items, demonstrating a well‐defined structure within this domain with a perfect replication index (RI = 1), indicating its high stability. Finally, Community 4 consists of items related to suicidal and self‐harming behaviours, also—with the exception of item 30—with excellent replication (RI = 1), underscoring its strong and consistent grouping.

Our results show partial consistency with these earlier proposals (Evans et al. 2002; Handscomb et al. 2016; Zeldovich and Alexandrowicz 2019). Specifically, while we identified meaningful clusters of items, the EGA revealed four distinct communities, suggesting that the structure may be more nuanced than recently (Aranyi et al. Under review; Zeldovich and Alexandrowicz 2019) assumed. These findings are paralleled by the work of Deng et al. (2024), who also identified a four‐factor CORE‐OM structure, although their grouping of items differed slightly from what was found in the present study.

Our study complements and contrasts with findings using EGA (Deng et al. 2024). Deng et al. identified a four‐factor structure that showed good fit indices. However, the factors identified in their study (general problems, positive resources, problems with others, and risk) differ from the communities observed in our study. For instance, while the risk scale in the study of Deng et al. comprised both risk to oneself and to others, the risk scale in our study was limited to risk to oneself. Discrepancies could be attributed to several factors, including differences in sample types (i.e. community help‐seeking sample vs. student sample) and their characteristics, and cultural contexts.

Of note, the ‘positive resources’ factor derived by Deng and colleagues subsumes all positive items within the instrument, while our EGA‐generated ‘positive resources’ scale only includes most of these items, while others are allocated to different latent constructs. This raises the question regarding a possible response‐effect (positive wording bias) that manifests in slightly superior indices of the Deng et al. model. Previous research indicated negative effects onto parameters of internal validity and reliability if mixed positive and negative items are included in one scale (Horan, DiStefano, and Motl 2003; Sonderen, Sanderman, and Coyne 2013).

The deviations from the three‐component model but also the inconsistency with Deng and colleagues' four‐factor model highlight the complexity in the search of a ‘true’ structure for the CORE‐OM. Despite methodological differences, the consistent emergence of complex structures across studies indicates that the CORE‐OM's factorial structure is indeed multi‐faceted and influenced by the specific analytical approaches employed. This raises a key issue in CORE‐OM research: Are we continually re‐deriving the most suitable structure through exploratory methods without fully grounding it in theoretical assumptions? While network analysis offers valuable insights, it may challenge the notion of a fixed, theoretically derived structure and instead highlight the fluidity of item relationships across samples and contexts.

In addressing the exploratory nature of our analysis, it is important to acknowledge that network analysis, while data‐driven, provides valuable insights into the relationships between individual CORE‐OM items, which may diverge from traditional theoretical models. This flexibility is advantageous, as it reveals nuances in item connections that might otherwise remain hidden. However, relying solely on such exploratory methods raises the risk of losing the tie to a solid theoretical foundation. Future research should aim to strike a balance between data‐driven exploration and the validation of findings within well‐established theoretical frameworks to ensure the robustness and clinical utility of the CORE‐OM in different language versions.

Our findings offer several implications for enhancing the scoring of the CORE‐OM. The identification of distinct communities through network analysis suggests a different factor structure than previously assumed. By aligning the scoring with these identified clusters—such as general problems, resources, interpersonal problems and self‐harming and suicidal behaviours—there is further potential to increase the accuracy of the instrument in capturing specific dimensions of psychological distress.

However, the variability in the reproducibility of the item‐cluster‐assignments suggests that not all identified group–item allocations are equally stable. This indicates that while refining subscales to reflect stable clusters like stress and suicidal behaviours may improve sensitivity, caution is needed with less reliable clusters. The revised factor structures offer a clearer framework for interpreting scores and aligning them with specific constructs, potentially leading to more accurate assessments and targeted interventions. Future revisions should focus on solidifying the less consistent clusters and integrating findings within well‐established theoretical frameworks to ensure robust and meaningful scoring.

Of note, the three‐factor and both four‐factor solutions showed very high model fits, if assessed via the WLSMV estimator, which is more appropriate for the assessment model relying on ordinally scaled indicators (DiStefano and Morgan 2014). However, based on MLM estimated AIC values, both EGA‐derived models are suggested as superior to previous models, while the model based on the results of Deng et al. (2024) provided the most parsimonious fit for our CFA sample. As they used the same EGA approach as our present study, applied to a Chinese student sample, their slightly better fit for clinical CORE‐OM data in an outpatient sample remains somewhat enigmatic at the moment and underlines the complexities and uncertainties bound to novel exploratory techniques like EGA and its transferability to traditional factor analytical techniques.

Nevertheless, the identification of central and predictable items within the network has important implications for improving the scoring of the CORE‐OM. Traditionally, all items are treated as equally contributing to the total score; however, our analysis reveals that certain items, such as Item 23 (Felt despairing or hopeless), have higher centrality and predictability, meaning they may play a more critical role in the assessment of overall psychological distress. Conversely, items like Item 8 (Troubled by aches/pains) show low predictability and influence, raising questions about their weighting in the total score.

These findings suggest that future revisions of the CORE‐OM could explore differential weighting of items based on their network characteristics, potentially leading to more precise scoring methods that better capture the core dimensions of distress and mental health.

### 4.1 Limitations and Future Directions

The cross‐sectional design limits conclusions on causality and temporal stability. Longitudinal studies are therefore needed to assess whether the item relationships and network structure remain stable across different clinical contexts or whether they fluctuate based on patient symptoms and therapeutic interventions. Furthermore, although network analysis offers novel perspectives, it is important to recognise that this method is largely data‐driven. The challenge remains to reconcile the insights from network analysis with theoretically grounded models, ensuring that any revisions to the CORE‐OM are based on both empirical evidence and clinical relevance.

Future research should also focus on examining whether bridge symptoms—items that connect different communities within the network—can provide useful insights into treatment pathways. Identifying these bridge items could highlight areas where interventions might have the most impact, by targeting symptoms that influence multiple aspects of psychological distress. In this regard, item‐level analyses are indicated to further contribute to the international applicability of the instrument and to provide a solid basis for cross‐national comparisons. For example, differential item functioning analyses using the item response theory framework would make it possible to identify groups (e.g., patients from different countries) with the same level of latent trait (e.g., symptom distress) who respond differently to certain items. These or similar analyses have been conducted for the Swedish (Åström, Sundström, and Lyrén 2023) and Russian CORE‐OM (Zeldovich, Ivanov, and Alexandrowicz 2019), but no cross‐translational comparisons have been published.

Given our findings, there is a compelling case for revising the CORE‐OM into a CORE‐OM‐R (revised version). The data suggests that certain items hold more centrality and predictability than others, offering a clearer understanding of symptom relationships. A revised version could incorporate these insights by refining the scoring system, perhaps through item weighting or even by reducing the number of items to focus on the most diagnostically useful indicators. Our results, particularly from the network analysis, provide an empirical basis for such a revision, as they reveal underlying patterns in item relationships that might not have been evident through traditional factor analysis.

## 5 Conclusions

Our results offer insights for refining the CORE‐OM's four‐domain scoring and highlight the potential benefits of developing a revised version that better reflects the relationships between items. The findings of the current network analysis and CFA partially align with previous research but also challenge some assumptions, suggesting a more complex and interconnected item structure than previously proposed. Future research should continue to integrate both data‐driven methods like network analysis and theoretical considerations to arrive at a more robust and clinically useful structure for the CORE‐OM.

## Ethics Statement

The studies involving human participants were reviewed and approved by the ethics board of the Sigmund Freud University Vienna, Austria. The participants provided their acceptance of the informed consent to participate in this study.

## Consent

All authors consented for the publication of the manuscript.

## Conflicts of Interest

The authors declare no conflicts of interest.

## Potential Identification of Study Participants

Not applicable.

## Supporting information


**Table S1** Sample descriptives.
**Table S2:** Item characteristics.
**Table S3.** Internal consistency of the CORE‐OM scales (original four domains and EGA‐derived solution).
**Figure S1:** Case‐dropping bootstrap technique to evaluate stability for the expected influence centrality, strength centrality and bridge centrality.
**Figure S2**: Visualisation of bootstraped confidence intervals of investigated edge weights in the network across 2000 bootstraps. The red line indicates the original edge weight values, the black line the bootstrap mean edge weight values and the grey‐shaded area the bootstrapped 95% CIs of the edge weight values.
**Figure S3**: Regularised partial correlation network (EBICglasso) of the CORE‐OM. *Note*: *N* = 2246; circles around the variables indicate explained variance (*R* ^2^).
**Figure S4**: Expected influence and strength centrality of investigated variables. *Note*: *N* = 2246.
**Figure S5**: The plot shows the differences between all pairs of expected influence. Each row and column represent a node. Black boxes represent significant differences between edge weights (*α* = 0.05). Grey boxes indicate nonsignificant differences.
**Figure S6**: The plot shows the differences between all pairs of centrality strength. Each row and column represent a node. Black boxes represent significant differences between edge weights (*α* = 0.05). Grey boxes indicate nonsignificant differences.
**Figure S7.** Replication indices regarding community detection of the individual items. *Note*: *N* = 2246.

## Data Availability

The raw data supporting the conclusions of this manuscript will be made available by the authors, without undue reservation, to any qualified researcher.
