# Supplementary material for: Uncovering the Internal Structure of the German Version of the CORE‐OM: A Network Analysis
Source: Clin Psychol Psychother. 2025 Mar 19;32(2):e70063. doi: 10.1002/cpp.70063 (PMC11923399; doi:10.1002/cpp.70063)
Supplement: Supplementary file 1 — Table S1 Sample descriptives. Table S2: Item characteristics. Table S3. Internal consistency of the CORE‐OM scales (original four domains and EGA‐derived solution). Figure S1: Case‐dropping bootstrap technique to evaluate stability for the expected influence centrality, strength centrality and bridge centrality. Figure S2: Visualisation of bootstraped confidence intervals of investigated edge weights in the network across 2000 bootstraps. The red line indicates the original edge weight values, the black line the bootstrap mean edge weight values and the grey‐shaded area the bootstrapped 95% CIs of the edge weight values. Figure S3: Regularised partial correlation network (EBICglasso) of the CORE‐OM. Note: N = 2246; circles around the variables indicate explained variance (R 2). Figure S4: Expected influence and strength centrality of investigated variables. Note: N = 2246. Figure S5: The plot shows the differences between all pairs of expected influence. Each row and column represent a node. Black boxes represent significant differences between edge weights (α = 0.05). Grey boxes indicate nonsignificant differences. Figure S6: The plot shows the differences between all pairs of centrality strength. Each row and column represent a node. Black boxes represent significant differences between edge weights (α = 0.05). Grey boxes indicate nonsignificant differences. Figure S7. Replication indices regarding community detection of the individual items. Note: N = 2246. [file CPP-32-e70063-s001.docx]

| **Table S1:** Sample descriptives. | | | | |  |  | | |
| --- | --- | --- | --- | --- | --- | --- | --- | --- |
|  |  | Sample EGA:  N = 2248 | | | Sample CFA:  N = 2248 | | | |
|  |  | Range | M | SD | Range | | M | SD |
| Age |  | 18 - 81 | 30.41 | 11.03 | 18 - 80 | | 31.01 | 10.89 |
|  | Category | Frequency | % | | Frequency | | % | |
| Gender | Female | 810 | 36 | | 792 | | 35.2 | |
|  | Male | 1396 | 62.1 | | 1417 | | 63 | |
|  | Divers | 42 | 1.9 | | 39 | | 1.7 | |
| Educational status | No degree | 37 | 1.6 | | 38 | | 1.7 | |
|  | Compulsory education | 219 | 9.7 | | 221 | | 9.8 | |
|  | Apprenticeship | 235 | 10.5 | | 240 | | 10.7 | |
|  | High school diploma | 599 | 26.6 | | 616 | | 27.4 | |
|  | Higher education | 888 | 39.5 | | 865 | | 38.5 | |
|  | Missing Data | 270 | 12 | | 268 | | 11.9 | |
| Origin | Germanophone | 1441 | 64.1 | | 1460 | | 64.9 | |
|  | Other^1^ | 807 | 35.9 | | 788 | | 35.1 | |
| Diagnosed with mental disorder | Yes | 1500 | 66.7 | | 1474 | | 65.6 | |
| Primary Diagnosis | F01-F09 | 1 | 0 | | 5 | | 0.2 | |
|  | F10-F19 | 20 | 0.9 | | 17 | | 0.8 | |
|  | F20-F29 | 19 | 0.8 | | 22 | | 1 | |
|  | F30-F39 | 487 | 21.7 | | 453 | | 20.2 | |
|  | F40-F48 | 837 | 37.2 | | 865 | | 38.5 | |
|  | F50-F59 | 29 | 1.3 | | 26 | | 1.2 | |
|  | F60-F69 | 85 | 3.8 | | 66 | | 2.9 | |
|  | F70-F79 | 1 | 0 | | 0 | | 0 | |
|  | F80-F89 | 5 | 0.2 | | 5 | | 0.2 | |
|  | F90-F98 | 16 | 0.7 | | 15 | | 0.7 | |
| Comorbidity | Yes | 588 | 26.1 | | 557 | | 24.8 | |
|  | | | | | | | | |

Notes. M: Mean, SD: Standard Deviation, F01-F09: Organic, including symptomatic, mental disorders

F10-F19: Mental and behavioral disorders due to psychoactive substance use, F20-F29: Schizophrenia, schizotypal, and delusional disorders, F30-F39: Mood (affective) disorders, F40-F48: Neurotic, stress-related, and somatoform disorders, F50-F59: Behavioral syndromes associated with physiological disturbances and physical factors, F60-F69: Disorders of adult personality and behavior

F70-F79: Intellectual disabilities, F80-F89: Disorders of psychological development, F90-F98: Behavioral and emotional disorders with onset usually occurring in childhood and adolescence. ^1^German was not the mother tongue, but sufficient language skills to answer the questionnaire.

**Table S2:** Item characteristics.

|  |  | Response patterns (%) | | | | | Characteristics | | | |
| --- | --- | --- | --- | --- | --- | --- | --- | --- | --- | --- |
| No. | Item (shortened version) | 0 | 1 | 2 | 3 | 4 | M | SD | SK | KU |
| 1 | Alone and isolated | 14.3 | 17.9 | 29.6 | 26.5 | 11.7 | 2.03 | 1.22 | -0.15 | -0.89 |
| 2 | Tense. anxious. nervous | 14.3 | 17.9 | 29.6 | 26.5 | 11.7 | 2.84 | 1.00 | -0.81 | 0.35 |
| 3 | Someone to turn to for support (+) | 23.4 | 24 | 26.9 | 17.6 | 8.1 | 1.63 | 1.24 | 0.25 | -0.95 |
| 4 | OK about myself (+) | 6.2 | 15.3 | 34.4 | 29.3 | 14.8 | 2.31 | 1.09 | -0.24 | -0.53 |
| 5 | Lacking in energy/enthusiasm | 7.3 | 15.1 | 25.6 | 31.2 | 20.6 | 2.43 | 1.18 | -0.39 | -0.72 |
| 6 | Physically violent to others | 93.2 | 4.2 | 1.5 | 0.7 | 0.4 | 0.11 | 0.46 | 5.29 | 31.59 |
| 7 | Able to cope when things go wrong (+) | 7.7 | 7.3 | 15.1 | 25.6 | 31.2 | 20.6 | 1.07 | -0.06 | -0.52 |
| 8 | Troubled by aches/pains | 23.6 | 19.3 | 22.6 | 20.7 | 13.8 | 1.82 | 1.37 | 0.10 | -1.21 |
| 9 | Thought of hurting myself | 70.9 | 12.2 | 8.9 | 5.6 | 2.3 | 0.56 | 1.02 | 1.80 | 2.27 |
| 10 | Talking too much for me | 14.1 | 16.2 | 31.1 | 27.9 | 10.7 | 2.05 | 1.20 | -0.21 | -0.82 |
| 11 | Tension/anxiety prevented | 14.4 | 16.4 | 70.9 | 12.2 | 8.9 | 5.6 | 2.3 | -0.22 | -1.00 |
| 12 | Happy with things done (+) | 5.6 | 19.8 | 38.1 | 28 | 8.5 | 2.14 | 1.01 | -0.12 | -0.43 |
| 13 | Disturbed. unwanted thoughts | 5.3 | 9.5 | 21.7 | 37.8 | 25.7 | 2.69 | 1.11 | -0.71 | -0.14 |
| 14 | Felt like crying | 11 | 14.7 | 24.6 | 31.5 | 18.2 | 2.31 | 1.24 | -0.38 | -0.82 |
| 15 | Felt panic or terror | 27.7 | 22.6 | 24.6 | 18.8 | 6.3 | 1.53 | 1.25 | 0.27 | -1.04 |
| 16 | Made plans to end my life | 82.5 | 9 | 5 | 2.7 | 0.9 | 0.31 | 0.76 | 2.77 | 7.44 |
| 17 | Overwhelmed by problems | 8.4 | 14.5 | 25.8 | 31.5 | 19.9 | 2.40 | 1.20 | -0.40 | -0.71 |
| 18 | 8.4 | 14.5 | 25.8 | 31.5 | 19.9 | 23.3 | 2.24 | 1.39 | -0.27 | -1.19 |
| 19 | Felt warmth/affection (+) | 21.4 | 28.3 | 25.2 | 16.1 | 9 | 1.63 | 1.24 | 0.33 | -0.86 |
| 20 | Problems impossible to put to one side | 6.5 | 14.1 | 25.3 | 35.4 | 18.8 | 2.46 | 1.14 | -0.46 | -0.55 |
| 21 | Done most things needed to (+) | 13.4 | 26.5 | 29.8 | 22.4 | 7.9 | 1.85 | 1.15 | 0.08 | -0.82 |
| 22 | Threatened/intimidated by someone | 88.2 | 6.1 | 3.4 | 1.6 | 0.8 | 0.21 | 0.65 | 3.63 | 13.74 |
| 23 | Felt despairing or hopeless | 10.5 | 16 | 25 | 30 | 18.5 | 2.30 | 1.24 | -0.33 | -0.86 |
| 24 | Better if dead | 58.6 | 15.1 | 12.1 | 8.5 | 5.7 | 0.88 | 1.24 | 1.22 | 0.23 |
| 25 | Felt criticised by others | 18.7 | 21.4 | 26.8 | 22.6 | 10.4 | 1.85 | 1.26 | 0.04 | -1.03 |
| 26 | Thought I have no friends | 38.7 | 19.3 | 18 | 15.1 | 8.9 | 1.36 | 1.36 | 0.54 | -1.00 |
| 27 | Felt unhappy | 5.3 | 11.9 | 24.8 | 35.1 | 22.8 | 2.58 | 1.12 | -0.54 | -0.42 |
| 28 | Images/memories disturbing | 19.1 | 16 | 22.3 | 25.9 | 16.8 | 2.05 | 1.36 | -0.15 | -1.19 |
| 29 | Irritable with other people | 19.8 | 23.5 | 27.5 | 20.1 | 9.2 | 1.76 | 1.24 | 0.14 | -0.97 |
| 30 | To blame for problems | 8.9 | 12.6 | 23 | 29.7 | 25.8 | 2.51 | 1.25 | -0.51 | -0.72 |
| 31 | Optimistic about future (+) | 8 | 17.8 | 33 | 27.2 | 14 | 2.21 | 1.13 | -0.18 | -0.67 |
| 32 | Achieved things wanted to (+) | 6.4 | 17.5 | 34.6 | 25.5 | 16 | 2.27 | 1.12 | -0.14 | -0.66 |
| 33 | Felt humiliated or shamed | 43 | 22.7 | 17.6 | 12.2 | 4.6 | 1.13 | 1.22 | 0.76 | -0.56 |
| 34 | Hurt self physically | 81.6 | 8.5 | 6 | 2.6 | 1.3 | 0.33 | 0.81 | 2.65 | 6.71 |
| Note. 0: not at all, 1: only occasionally, 2: sometimes, 3: often, 4: most or all the time, M: mean, SD: standard deviation, SK: skewness, KU: kurtosis. | | | | | | | | | | |

**Table S3.** Internal consistency of the CORE-OM scales (original four domains and EGA-derived solution)

| Model | No. of items. | α | ω |
| --- | --- | --- | --- |
| Original four domains (Evans et al., 2002) | | | |
| Functioning | 12 | .80 | 0.54 |
| Problems/Symptoms | 12 | .86 | 0.76 |
| Well-Being | 4 | .68 | 0.57 |
| Risk | 6 | .74 | 0.65 |
| EGA-derived four-factor (present study) | | | |
| General Problems | 15 | 0.90 | 0.79 |
| Interpersonal Problems | 8 | 0.65 | 0.40 |
| Positive Resources | 6 | 0.83 | 0.78 |
| Self-Harm Risk | 5 | 0.72 | 0.65 |
| EGA-derived four-factor (Deng et al. 2024) |  |  |  |
| General Problems | 15 | 0.89 | 0.79 |
| Risk | 6 | 0.74 | 0.66 |
| Positive Resources | 8 | 0.80 | 0.70 |
| Problems with Others | 5 | 0.71 | 0.58 |
| Note. No. of items in a scale, α: Cronbach’s α, ω: McDonald’s ω, 95%CI: 95% confidence interval. N = 4496 | | | |


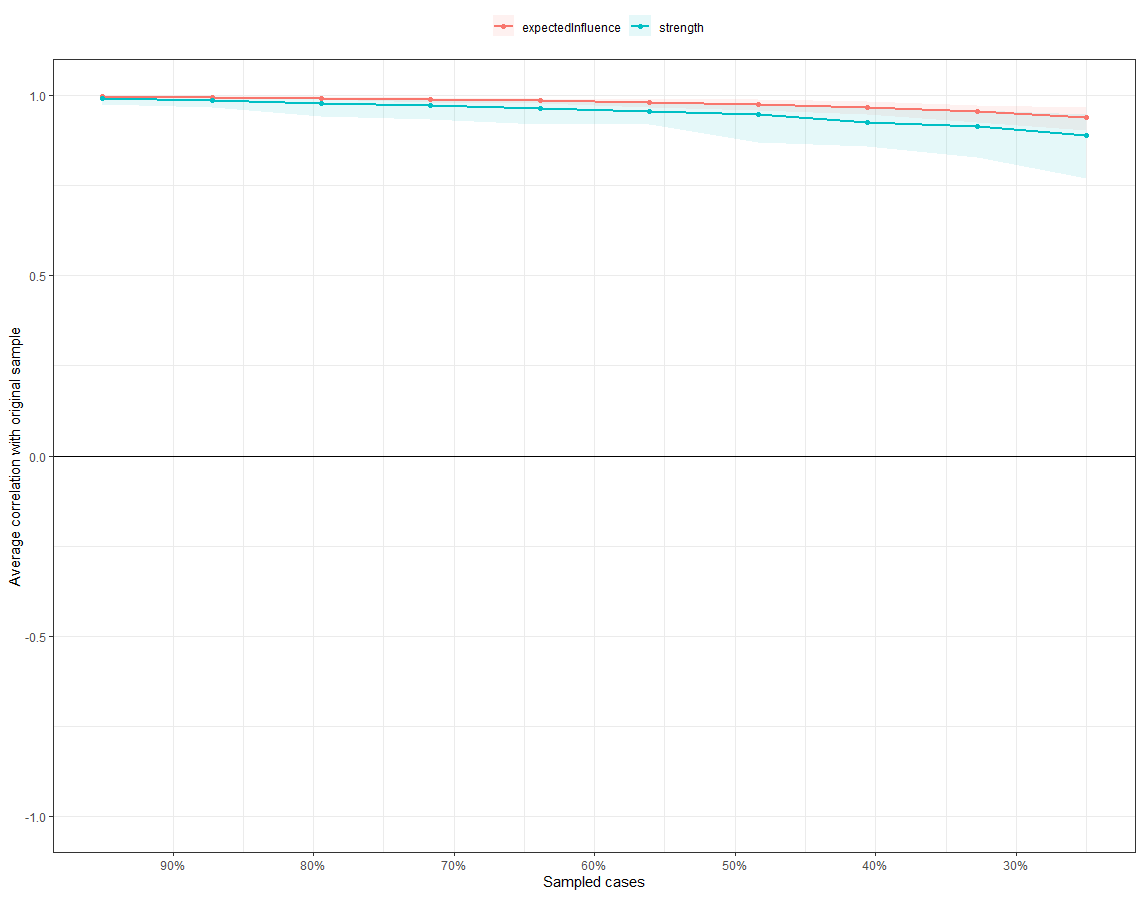


**Figure S1:** Case-dropping bootstrap technique to evaluate stability for the Expected Influence centrality, Strength centrality and Bridge centrality.


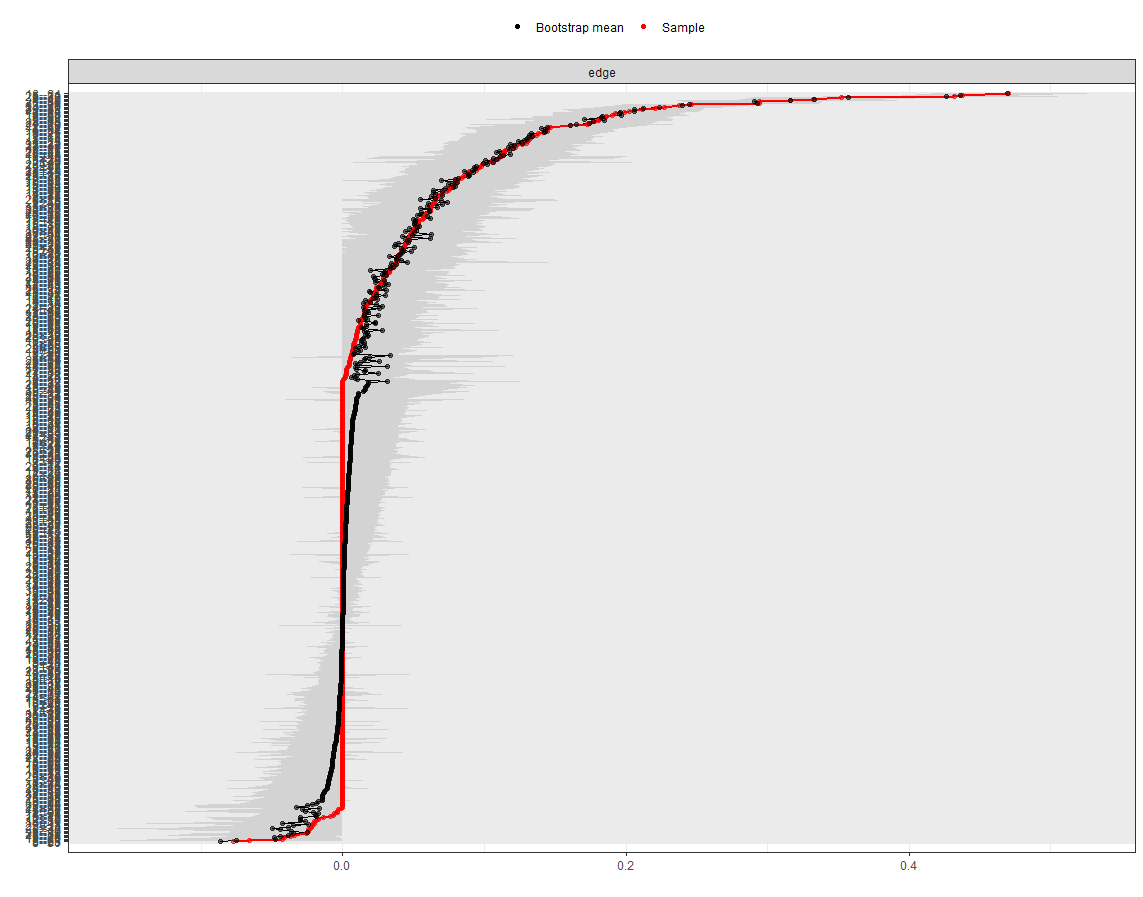


**Figure S2**: Visualization of bootstraped confidence intervals of investigated edge weights in the network across 2000 bootstraps. The red line indicates the original edge weight values, the black line the bootstrap mean edge weight values and the gray-shaded area the bootstrapped 95% CIs of the edge weight values.


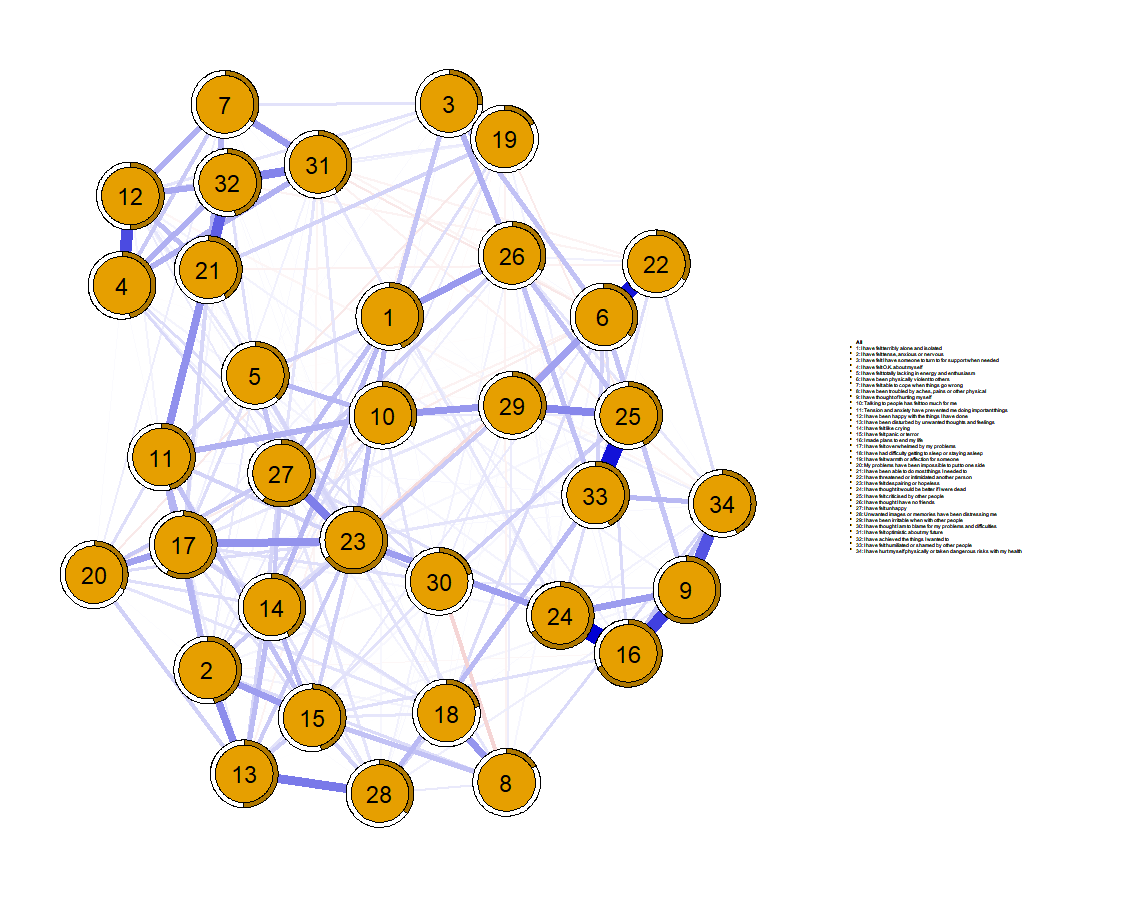


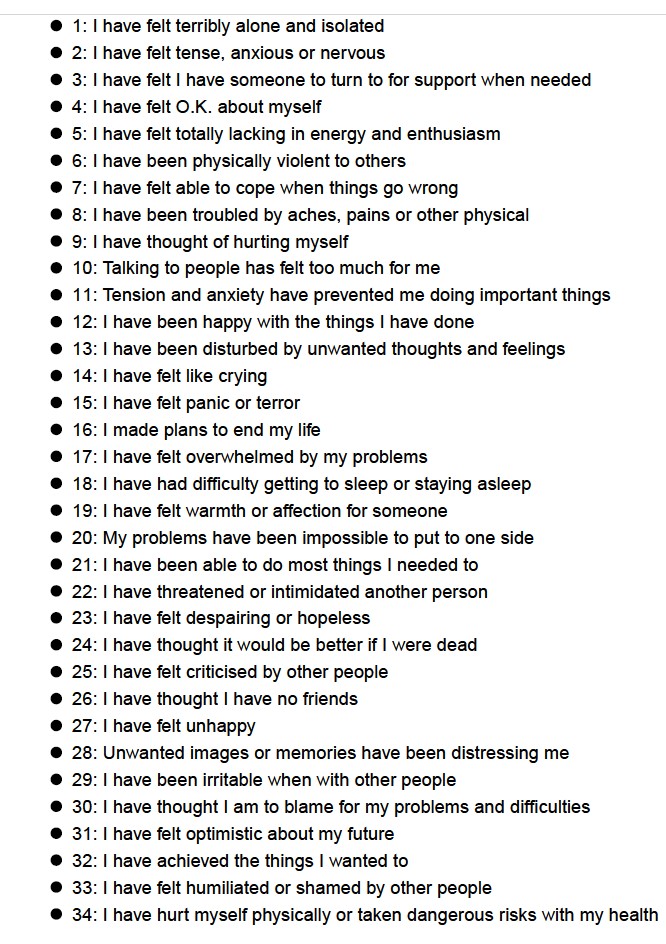


**Figure S3**: Regularized partial correlation network (EBICglasso) of the CORE-OM.

Note. N = 2246; circles around the variables indicate explained variance (R²).


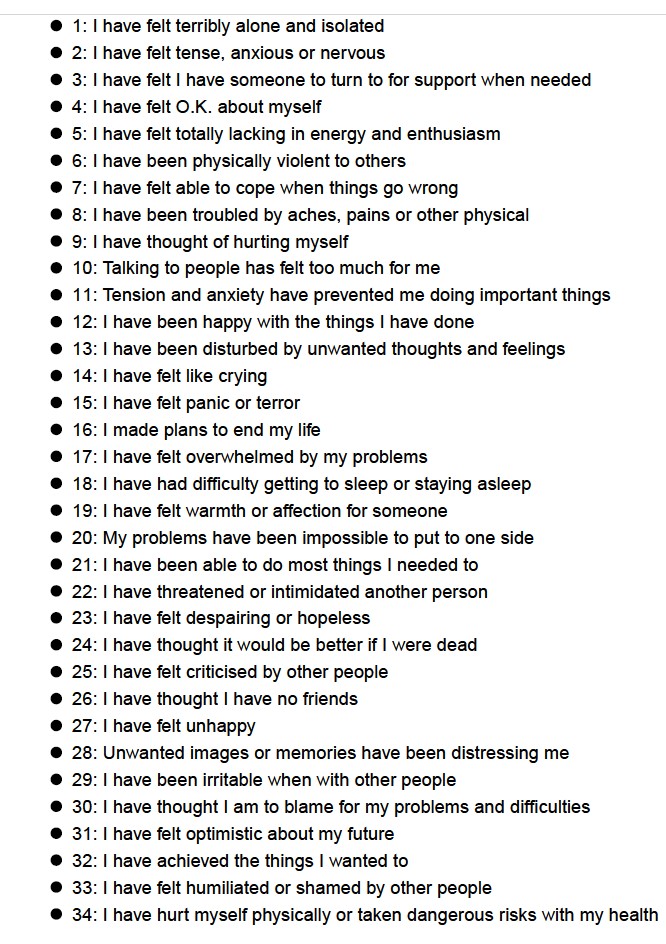

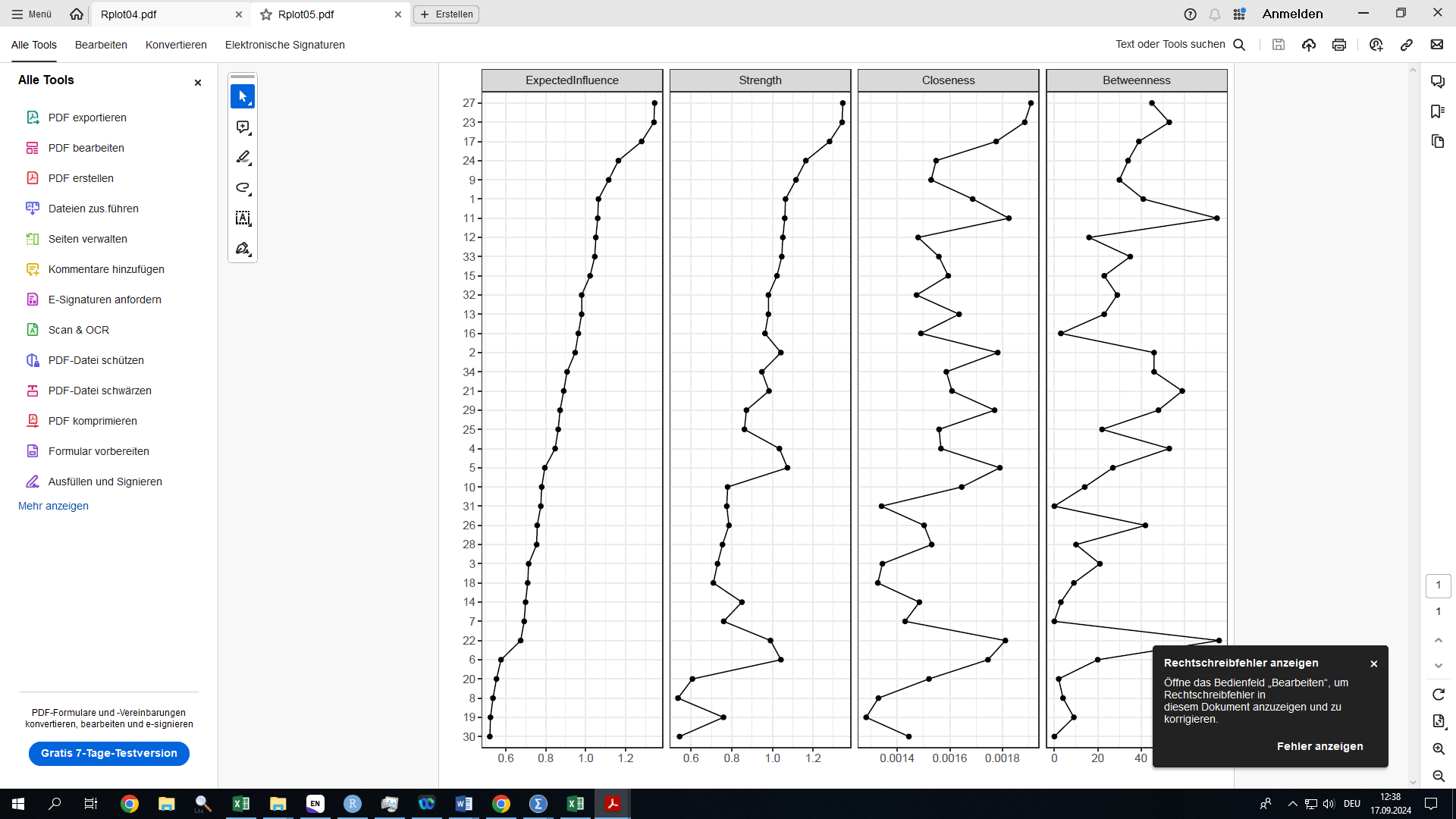


**Figure S4**: Expected influence and strength centrality of investigated variables.

Note. N = 2246.


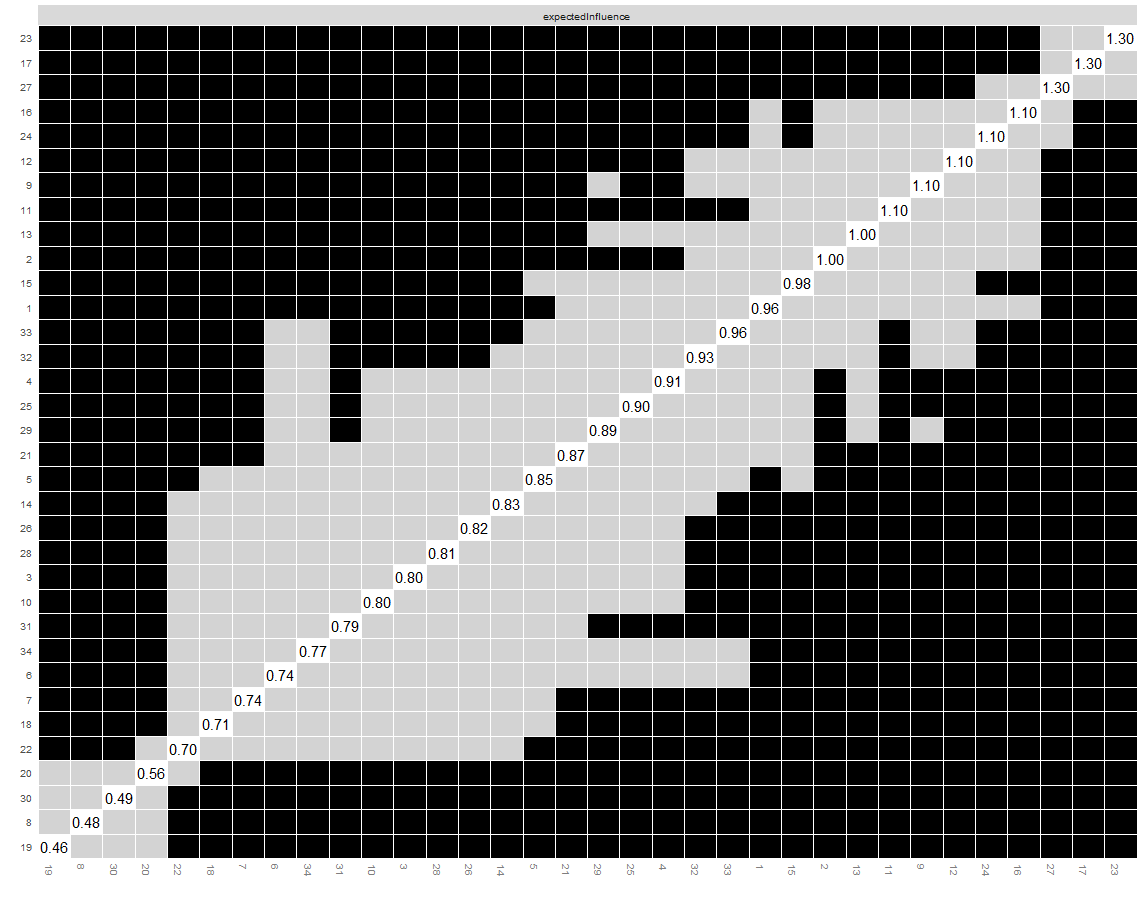


**Figure S5**: The plot shows the differences between all pairs of expected influence. Each row and column represent a node. Black boxes represent significant differences between edge weights (α = .05). Gray boxes indicate non-significant differences


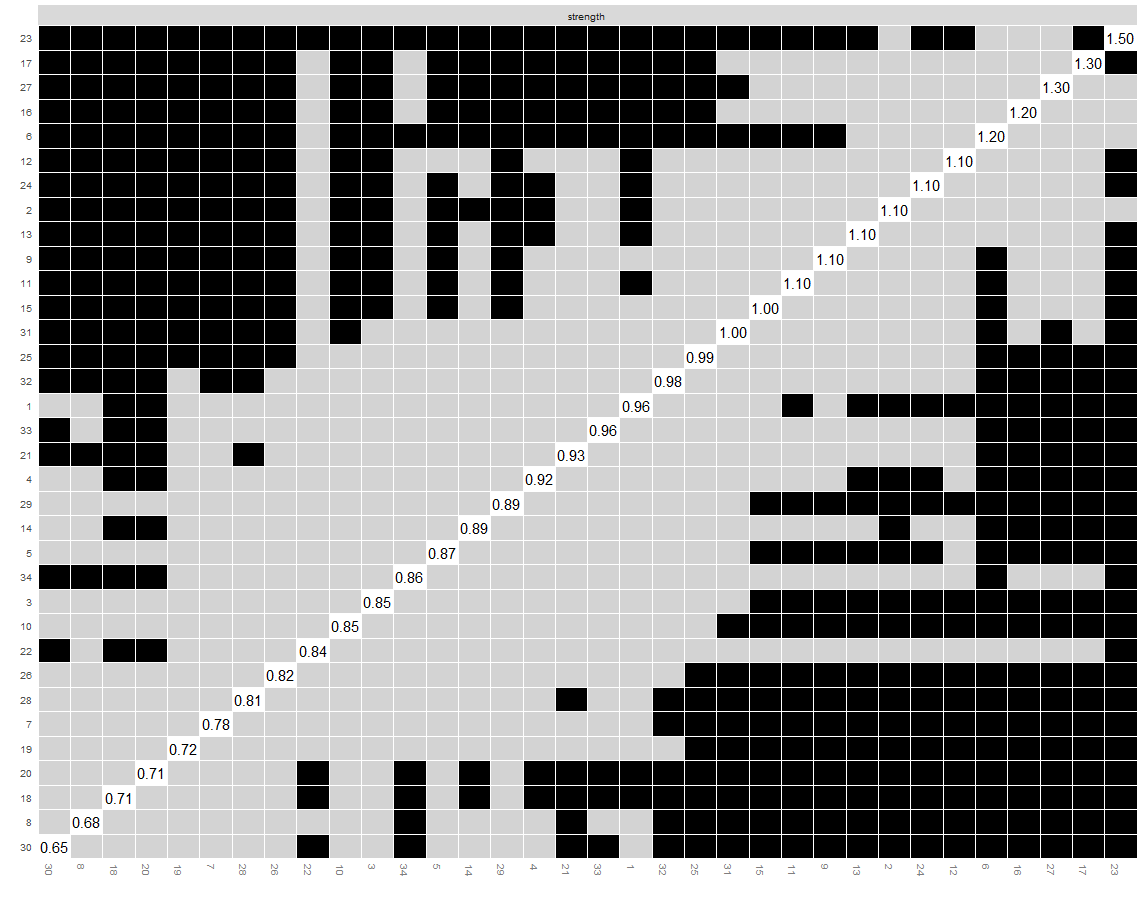


**Figure S6**: The plot shows the differences between all pairs of centrality strength. Each row and column represent a node. Black boxes represent significant differences between edge weights (α = .05). Gray boxes indicate non-significant differences


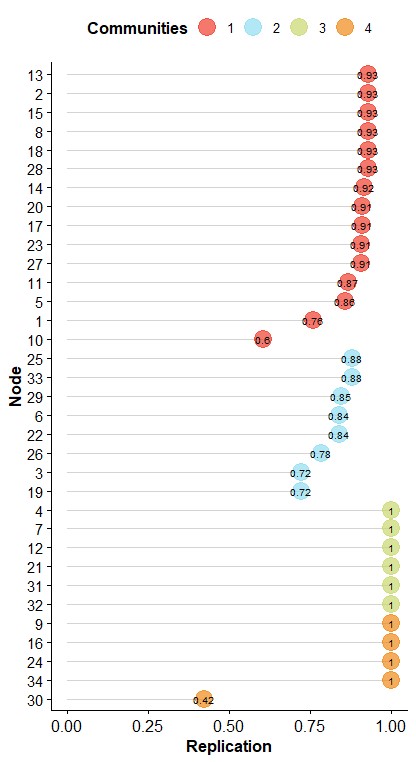


**Figure S7**. Replication indices regarding community detection of the individual items.

Note. N = 2246.
